# Bichloride of Mercury in Dental Practice

**Published:** 1892-03

**Authors:** George S. Allan

**Affiliations:** New York


### BICHLORIDE OF MERCURY IN DENTAL PRACTICE.[1]

BY DR. GEORGE S. ALLAN, NEW YORK.

As to my employment of mercury bichloride, I would say that for some years past I have used it more freely than is customary in the profession, and with much satisfaction.

There is little doubt in my mind that it is one of the most powerful germicides we possess; that its use is not attended with any special dangers, and that most of the objections that have been urged against it are largely fallacious and misleading, and due to a lack of knowledge as to its properties and combinations.

In the last year there have been several notable articles in the journals relating to the subjects of infection, disinfection, sepsis, asepsis, and sterilization of dental instruments. They all lay stress on the importance of the subject, and the great danger that a lack of proper care and attention may entail. Their general drift may be summed up in brief: that sterilization of instruments, etc., is necessary, and, in a measure, obligatory; but that ways and means are not easily attainable, and in some cases not at all.

Dry heat is the most efficacious means to employ to destroy germs, and they fall back on the oven at about 300° F. as the best attainable method to adopt.

To all this I have no special objections to offer other than the manifest one, that it does not meet our wants. Dry heat will

---

[1] Remarks made at the Twenty-fourth Annual Meeting of the American Academy of Dental Science, Boston, November 11, 1891.

destroy germs most assuredly; but who can afford to keep his instruments in the oven half the time, or give it the requisite care and attention? Who can afford the large number of duplicates required where certain instruments are not only in daily but hourly use, and are picked up, maybe, a dozen times or more a day, let alone the complete failure of said means to meet the case of the mouth-mirror, syringe, etc.?

What the dentist wants is something always ready, available, and efficient. If he cannot stop to cook his instruments, he can take the time to wipe them clean and dip them into a cup or dish containing a germ-destroying fluid.

I have, then, mercuric bichloride on my shelf in a bottle, containing a one-per-cent. solution. When wanted, I prepare from this a solution of the proper strength, one to one thousand; but instead of water I take rose-water. The bichloride solution simply has a very disagreeable taste; but if a mixture is made of one-per-cent. solution bichloride and rose-water, in proportion of one of the former to nine of the latter, you have an equally efficient preparation, and the nasty bug-poison taste of the simple liquid is supplanted by an agreeable rose-flavored one. This, then, is the mixture the use of which I advocate.

Into this fluid I freely dip any or all of my instruments, and as freely pass them into the mouth and around the teeth and gums. The instruments are never injured by the operation, and are easily kept clean. All instruments that have been dipped into water and wiped clean and dry can have only a thin layer of germs attached to them, and these will be reached at once by the solution and destroyed. I also constantly dip my mouth-mirror into the same fluid, and find that it is in no way injured by it; on the contrary, all parts of it are kept the brighter and sweeter. My syringe, a metal one, is also kept in good condition by its use, and I find now that patients, instead of asking me what the nasty stuff I have in the glass is that I have washed their teeth with, wonder where the delightful rose-taste comes from.

In cleaning teeth I dip the steel scalers of all descriptions and sizes constantly in the fluid, and then apply them to the necks of the teeth, and feel certain it is a beneficial operation. Infection can be carried from one part of the mouth to another, but by this means all danger is averted.

I have repeatedly noticed the rapid and healthy healing of the gums and soft tissues, after extended and even painful lacerations, resulting from this operation.

The instruments I exhibit—scalers, scrapers, mouth-mirror, and syringe—have been dipped into the solution hundreds of times, and, as you see, show no indications of other than ordinary use.

I would add that the rose-water solution is equally valuable in case you desire to cleanse and wash out a pocket or an abscess, and your patients will thank you for giving them so agreeable a taste in their mouths, instead of the ordinary disagreeable one.

In preparing the solution, you must not forget that plain bichloride decomposes; and even if you employ a freshly-prepared solution, you fail to get the best effects, owing to the albumen-coagulating power of the agent. Common salt in equal proportions with the bichloride is efficient; but probably the tartacid sublimate tablets are about the most quickly available and efficient condition in which to keep the article. One of these tablets mixed with a pint of water makes a one to two thousand solution. Apparently this solution does not change, and does not coagulate albumen.

Another tablet is composed of ammoniac chloride and mercury bichloride in equal proportions.

